# Family Medicine for internally displaced persons in Mali: A training of trainers approach

**DOI:** 10.4102/phcfm.v17i1.4826

**Published:** 2025-03-12

**Authors:** Drissa M. Sidibé, Ann Isabelle Grégoire, Véronique Lisée, Pierre Rodrigue Traoré, Inhissa B. Bengaly, Aboubakary Konaté, Ibrahim H. Sidibé, Sabina Abou Malham, David-Martin Milot, Gabriel Blouin-Genest

**Affiliations:** 1 Department of Family Medicine & Community Medicine, Faculty of Medicine, Pharmacy and Odonto-Stomatology, University of Sciences, Techniques and Technologies of Bamako, Bamako, Mali; 2 University Community Health Center of Banconi, Ministry of Health and Public Hygiene, Bamako, Mali; 3 Department of Family Medicine and Emergency Medicine, Faculty of Medicine and Health Sciences, Université de Sherbrooke, Sherbrooke, Canada; 4 Centre Interdisciplinaire De développement International en Santé (CIDIS), Faculty of Medicine and Health Sciences, Faculty of Arts and Humanities, Université de Sherbrooke, Longueuil, Canada; 5 Center for Health Sciences Pedagogy (CPSS), Faculty of Medicine and Health Sciences, Université de Sherbrooke, Sherbrooke, Canada; 6 Department of Regional Health Directorate, Ministry of Health and Public Hygiene, Kayes, Mali; 7 National Office for Reproductive Health, Ministry of Health and Social Development, Bamako, Mali; 8 International Association for Maternal and Neonatal Health (IAMANEH) Suisse, Bamako, Mali; 9 School of Nursing Sciences, Faculty of Medicine and Health Sciences, Université de Sherbrooke, Longueuil, Canada; 10 Charles Lemoyne Research Center, Faculty of Medicine and Health Sciences, Université de Sherbrooke, Longueuil, Canada; 11 Department of Community Health Sciences, Faculty of Medicine and Health Sciences, Université de Sherbrooke, Sherbrooke, Canada; 12 School of Applied Political Science, Faculty of Arts and Humanities, Université de Sherbrooke, Longueuil, Canada

**Keywords:** family medicine, primary health care, decentralised training, internally displaced persons, humanitarian crisis, co-construction, cascade training, multidisciplinary

## Abstract

Mali is currently experiencing a polycrisis, characterised by the interplay of growing socio-political insecurity, massive population displacements and recurrent tensions in the functioning of the health system and the provision of care. In this context, the multidisciplinary teams of University Community Health Centres (CSCoM-Us), primary health care facilities, have expressed the desire to strengthen their skills to better meet the needs of the internally displaced persons who frequent their facilities. To address this demand, training workshops were co-constructed by a team of family physicians (FPs), combining the experiential knowledge of local teams with the expertise of partners from the *Université de Sherbrooke*. A Training of Trainer (ToT) programme, consisting of training provided by central-level trainers to local-level practitioners, was developed and deployed. Five priorities were identified by local partners: continuity of care, maternal health, gender-based violence, mental health and working with a language barrier. From 2022 to 2023, this training was implemented in Mali’s seven CSCOM-Us, reaching 277 health professionals in five regions of the country. The outcomes include increased awareness of the challenges faced by internally displaced persons and strengthening local capabilities. This short report highlights the strategic role and leadership played by FP in improving the population’s health, particularly in sub-Saharan Africa, through their versatility and community-oriented, holistic and patient-centred approach.

## Introduction

Mali is among the countries with the lowest rates of physicians per capita, and approximately 0.1 physician per 1000 inhabitants.^1^ The specialty of Family Medicine & Community Medicine (FM & CM) was introduced in 2012 at the Faculty of Medicine and Odontostomatology (FMOS) of the University of Sciences, Techniques and Technologies in Bamako, thanks to the DECLIC project (*Projet d’appui à la formation des professionnels de la santé au Mali*), a collaboration between Mali and Canada (2010–2018, 19.1 million CAN$, funded by Global Affairs Canada). By 2024, 75 Malian doctors, including 20 women, had obtained their FM & CM diploma and were practising in various regions and health facilities throughout the country.

In the context of a national shortage of health professionals, and in line with the World Health Organization’s strategies for ‘integrated, people-centred services’,^2^ one of the pillars of the FM & CM diploma is the decentralised residency training within a network of University Community Health Centres (CSCom-Us). These primary health care facilities, located in communities, are spread across five regions in Mali: Bamako, Kayes, Koulikoro, Ségou and Sikasso.^3^ The goal of the programme is to train FP who specialise in working at the primary level of the pyramid of care, focusing on multidisciplinary work, preventative and curative care, while targeting the social determinants of health.^4^

In Mali’s current polycrisis context, family physicians (FPs) are key leaders who can act as agents of change in responding to existing and emerging needs of communities.^5^ This short report presents an innovative Training of Trainer (ToT) programme codesigned with Malian FPs to meet the health needs of internally displaced persons (IDPs). The report begins by highlighting the key health challenges currently affecting Mali, followed by a detailed description of the methodology and concludes with the presentation of outcomes and key recommendations.

## The changing socio-political context in Mali and the impact on primary health care

Since 2012, Mali has been facing growing socio-political insecurity that is undermining social, health and education services.^6^ Women and children are the first victims of these crises, also suffering from the underfunding of the health system in a country ranked 141st out of 146 in the Global Gender Gap Report^7^ and 188th out of 193 in the Human Development Index.^8^ The deteriorating security situation in northern Mali is gradually spreading to the central and southern regions, with devastating consequences for the civilian population, including large-scale population displacement. According to the International Organization for Migration (IOM), there were at least 331 000 IDPs and 94 000 refugees in Mali in 2024.^9^

At the health level, Mali faces a high burden of communicable diseases (CDs), including malaria, tuberculosis and HIV/AIDS. In 2021, 7.7 million cases of malaria were reported resulting in almost 20 000 deaths, while the mortality rate from tuberculosis has remained at 7.5 per 100 000 population from 2015 to 2021. In addition, approximately 56 194 people were receiving anti-retroviral treatment for HIV in 2021. In recent years, non-communicable diseases (NCDs) (such as cardiovascular diseases, chronic respiratory diseases, cancer and diabetes) have also emerged as significant contributors to the country’s disease burden.^10^

The ongoing polycrisis in Mali exacerbates the disease burden in several ways. Internally displaced persons, living in overcrowded host families or shelters with limited access to basic services such as food, clean water and sanitation, face heightened risks of both CDs and NCDs. Violent attacks on civilians and recurrent gender-based violence (GBV) contribute to physical and mental trauma. As seen in other humanitarian crises, disruptions in the healthcare system significantly impact access, continuity and quality of care, leading to a deterioration in basic health conditions and a decline in childhood immunisation. For instance, the measles immunisation rate in Mali was 73% in 2023.^11,12,13^

Consequently, health professionals in primary health care facilities, particularly CSCom-Us, are increasingly called upon to intervene with the displaced populations. However, these patients present complex needs, and many feel inadequately trained to respond effectively. In this context, a group of Malian FP has designed a ToT programme as part of the *Communautés locales d’enseignement pour des femmes et des filles en santé* (CLEFS) project, aiming to support agents of change in providing comprehensive and more effective care to IDPs.

## Mobilising family physician to respond to the internally displaced person crisis: A method for the training of trainer model and its evaluation

The objective of this project was to co-construct a training programme and deploy it through key strategic locally designed interventions. The ToT model^14,15^ was adopted as the methodological approach for this intervention. This approach facilitates rapid capacity building and knowledge dissemination, making it particularly effective in low-resource settings. Within this model, an expert trainer provides instruction to a cohort of trainees, who subsequently disseminate the acquired knowledge and skills within their respective health centres. It is designed to promote local ownership and ensure sustainable impact.

The model’s cost-effectiveness was recently demonstrated in a recent systematic review of ToT programmes implemented in low- and middle-income countries.^14^ The findings also underscored the critical role of co-construction and active involvement of local stakeholders in designing the training, ensuring its adaptation to the specific cultural and contextual needs of the target population.

The ToT model employed in this intervention was complemented by a participatory learning approach, in which the learners – health professionals – played an active role in shaping the content and focus of the training. This approach facilitated a more tailored and contextually relevant learning experience. In 2022, a training need prioritisation survey was distributed via email to healthcare professionals (family physicians, nurses and midwives) working across the seven CSCOM-Us in Mali. A total of 17 responses were collected. The programme design team, composed of five Malian FP with extensive clinical and teaching roles in their facilities, in collaboration with three partners from the Université de Sherbrooke with expertise in refugee health and health education, identified priorities after analysing the survey and consulting evidence-based best practices regarding health of people experiencing forced displacement.^12^

A participatory approach was also employed during the training of the first cohort of trainees, working in small groups and asking each participant to share ideas to develop core recommendations, which they would later teach at their respective health centres. This approach encourages active engagement from all participants in generating solutions. Although individual change was not quantitatively assessed through pre- and post-training questionnaires, participant satisfaction with the pedagogical tools employed was evaluated using a post-training evaluation survey.

## Processes and results

Topics identified as priorities in the context of care for displaced persons in the Malian context included continuity of care, maternal health, GBV, mental health and working with a language barrier.

Five modular workshops were created, each corresponding to one of the identified themes. Training content was adapted from published guidelines^16,17,18,19,20,21,22^ to the local setting by the five Malian FP part of the programme design team. The training was designed to encourage learners to better understand the differences in terms of needs between IDPs and the populations normally served by CSCom-Us, and then to adapt healthcare services to better meet the specific needs of IDPs.

**FIGURE 1:**
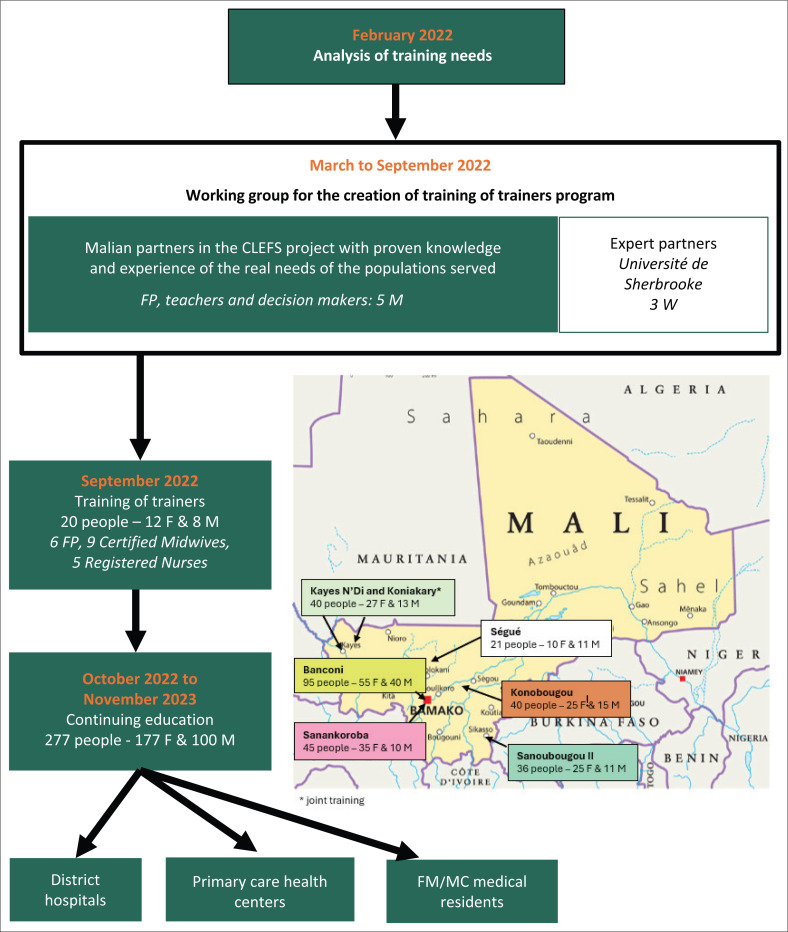
Sidibé DM, Grégoire A I, Lisée V. et al. 2024. ToT model, including training locations and number of professionals reached.

An initial 2-day training for trainers was held in Bamako in September 2022 with 12 participants, including doctors, nurses and midwives. At the end of the training sessions and interactive workshops, key recommendations for the care of IDPs in Mali’s CSCOM-Us emerged, highlighting the experiential knowledge of participants in the field. The training was then extended to the seven CSCom-Us in Mali between 2022 and 2023, allowing knowledge transfer to five regions of the country and reaching different categories of health professionals, such as doctors, nurses, midwives, assistants, as well as residents and interns (Figure 1).

## Impact and future challenges

Although co-development between actors from different practice backgrounds was challenging, evaluation surveys gathered from participants revealed a heightened awareness of the challenges faced by IDPs, both at the medical and psychosocial levels, and appreciation of the quality and diversity of pedagogical methods. The development of the training also led to the creation of a community of practice. This dynamic community of doctors, nurses and midwives is helping to strengthen clinical capacities of frontline resources, as well as local collaborative and transformational leadership in crisis-affected regions.

Future challenges include the adoption of the training by local actors as well as regional and ministerial decision-makers to ensure the sustainability and wider dissemination of the project. The evaluation of the mid- and long-term impact of the project will be key to a better regional and contextual adaptation in the future.

The training modules for IDPs care should also be adopted by medical institutions to ensure adequate guidance and awareness as part of the initial academic programme of future health professionals. Finally, while no specific strategies were implemented to address gender disparities in training access during this intervention, this issue should be prioritised in future efforts.

## Conclusion

At a time when more and more stakeholders are emphasising the importance of family medicine in providing equitable and quality primary health care,^23^ this short report illustrates how FP can play a critical role in continuously improving the health of communities, especially those displaced and affected by crisis. Through a bottom-up, patient-centred, holistic and multidisciplinary approach deeply rooted in the community, FP emphasise prevention and integrate the social determinants of health in their care. In Mali, as elsewhere, it is critical to continue recognising and value the unique role of FP as local leaders in addressing the country’s current challenges.

The authors would like to acknowledge all Malian partners for their commitment to this project and their communities. The authors would like to thank Michèle Rietmann, Johannie Lapierre, Mahamane M. Maïga and Sarah Stecko for their support in writing this short report. The authors would also like to thank the Faculty of Medicine and Odontostomatology (FMOS) of the University of Sciences, Techniques and Technologies in Bamako, as well as the Université de Sherbrooke, Cégep de Saint-Jérôme, and Centre de Coopération internationale en Santé et Développement (CCISD), now Santé Monde in Canada.

The authors reported that they received funding from Global Affairs Canada, which may be affected by the research reported in the enclosed publication. The authors have disclosed those interests fully and have implemented an approved plan for managing any potential conflicts arising from their involvement. The terms of these funding arrangements have been reviewed and approved by the affiliated university in accordance with its policy on objectivity in research.

This work was supported by the Communautés locales d’enseignement pour des femmes et des filles en santé (CLEFS) project, which is funded by Global Affairs Canada (Project number: P006775).

The authors confirm that the data supporting the findings of this study are available within the article, on the following website ‘www.cidis.ca’ or from the corresponding author, A.I.G., on reasonable request.
